# Valganciclovir—Ganciclovir Use and Systematic Therapeutic Drug Monitoring. An Invitation to Antiviral Stewardship

**DOI:** 10.3390/antibiotics10010077

**Published:** 2021-01-15

**Authors:** Alicia Galar, Maricela Valerio, Pilar Catalán, Xandra García-González, Almudena Burillo, Ana Fernández-Cruz, Eduardo Zataráin, Iago Sousa-Casasnovas, Fernando Anaya, María Luisa Rodríguez-Ferrero, Patricia Muñoz, Emilio Bouza

**Affiliations:** 1 Clinical Microbiology and Infectious Diseases Department, Hospital General Universitario Gregorio Marañón, Doctor Esquerdo 46, 28007 Madrid, Spain; mavami_valerio@yahoo.com.mx (M.V.); pilar.catalan@salud.madrid.org (P.C.); almudena.burillo@gmail.com (A.B.); anafcruz999@gmail.com (A.F.-C.); emilio.bouza@gmail.com (E.B.); 2 Instituto de Investigación Sanitaria Gregorio Marañón, 28007 Madrid, Spain; xandra.garcia@salud.madrid.org; 3 Pharmacy Department, Hospital General Universitario Gregorio Marañón, 28007 Madrid, Spain; 4 Cardiology Department, Hospital General Universitario Gregorio Marañón, 28007 Madrid, Spain; eduzatanico@gmail.com (E.Z.); iagosousa@yahoo.es (I.S.-C.); 5 Nephrology Department, Hospital General Universitario Gregorio Marañón, 28007 Madrid, Spain; fanayaf@senefro.org (F.A.); mlrodriguezf@senefro.org (M.L.R.-F.); 6 CIBER de Enfermedades Respiratorias (CIBERES CB06/06/0058), 28007 Madrid, Spain; 7 Medicine Department, School of Medicine, Universidad Complutense de Madrid, 28007 Madrid, Spain

**Keywords:** valganciclovir, ganciclovir, serum levels, therapeutic drug monitoring, CMV infection

## Abstract

Valganciclovir (VGCV) and ganciclovir (GCV) doses must be adjusted according to indication, renal function and weight. No specific therapeutic exposure values have been established. We aimed to evaluate the adequacy of VGCV/GCV doses, to assess the interpatient variability in GCV serum levels, to identify predictive factors for this variability and to assess the clinical impact. This is a prospective study at a tertiary institution including hospitalized patients receiving VGCV/GCV prophylaxis or treatment. Adequacy of the antiviral dose was defined according to cytomegalovirus guidelines. Serum levels were determined using High-Performance Liquid Chromatography. Blood samples were drawn at least 3 days after antiviral initiation. Outcome was considered favorable if there was no evidence of cytomegalovirus infection during prophylaxis or when a clinical and microbiological resolution was attained within 21 days of treatment and no need for drug discontinuation due to toxicity. Seventy consecutive patients [74.3% male/median age: 59.2 years] were included. VGCV was used in 25 patients (35.7%) and GCV in 45 (64.3%). VGCV/GCV initial dosage was deemed adequate in 47/70 cases (67.1%), lower than recommended in 7/70 (10%) and higher in 16/70 (22.9%). Large inter-individual variability of serum levels was observed, with median trough levels of 2.3 mg/L and median peak levels of 7.8 mg/L. Inadequate dosing of VGCV/GCV and peak levels lower than 8.37 or greater than 11.86 mg/L were related to poor outcome. Further studies must be performed to confirm these results and to conclusively establish if VGCV/GCV therapeutic drug monitoring could be useful to improve outcomes in specific clinical situations.

## 1. Introduction

Ganciclovir (GCV), a nucleoside analog that targets DNA polymerase, is indicated for the prophylaxis and treatment of cytomegalovirus (CMV) related infections [1,2,3,4,5] and is mostly administered as an intravenous formulation due to its low oral bioavailability. Valganciclovir (VGCV) is a GCV ester that is well absorbed after oral administration and is rapidly metabolized to GCV. The pharmacokinetics of GCV varies according to different parameters, including renal function and weight [1,4,6,7,8,9,10]. However, clinical studies are scarce, partial and usually applied to specific populations [4,6,10].

GCV trough and peak serum levels have not been properly associated with clinical efficacy [11,12,13,14], the utility of therapeutic drug monitoring (TDM) for patients receiving VGCV and GCV remains unclear and TDM is not routinely indicated by institutions and international consensus guidelines [15,16,17]. However, some studies suggest that GCV exposure might be suboptimal in pediatric and adult solid organ transplant (SOT) recipients treated with standard doses of VGCV [10] and that TDM might be useful in patients with fluctuating renal function and/or not responding to treatment as expected, and to minimize the risk of drug-related hematological toxicity particularly in hematologic stem cell transplant (HSCT) recipients [17].

Antimicrobial stewardship programs are an important part of policies to enhance patient safety and are considered a cost-effective approach for optimizing the antimicrobials use [18]. In routine clinical practice, VGCV and GCV doses are prescribed according to the patients’ weight, renal function and clinical indication (prophylaxis or treatment) [19], but literature is scarce regarding optimal strategies to prevent CMV infection in different populations [20,21] and dosing compliance according to guidelines.

The objectives of this study were to evaluate the adequacy of VGCV/GCV prescription according to international recommendations in a non-selected inpatient population, to assess the interpatient variability in GCV serum levels, to identify the potential predictive factors for this variability and to assess the clinical impact of the different ganciclovir serum levels.

## 2. Material and Methods

### 2.1. Study Design

This is a prospective observational study performed at a tertiary-care hospital including consecutive hospitalized patients receiving VGCV or GCV for prophylaxis or treatment, who accepted participation by signing an informed consent provided by one of the researchers.

VGCV or GCV treatment and dosage were decided by the attending physicians. The antiviral dose was afterward classified by the authors as adequate, low or high, according to CMV international guidelines [19]. Doses were adjusted to renal function. Adequate dose of treatment in patients with normal renal function was VGCV 900 mg/12 h or GCV 5 mg/kg/12 h. Normal dose for prophylaxis was VGCV 900 mg/24 h or GCV 5 mg/kg/24 h.

On SOT and hematologic stem cell transplant (HSCT) recipients, the local protocols of prophylaxis, preemptive therapy and treatment were followed and CMV viral load was determined as recommended for each scenario.

CMV viral load was measured in all included patients on a weekly basis, at least till its negativity. After that, it was repeated at the discretion of the treating physician and depending on the patient underlying condition.

GCV serum levels were determined by HPLC (High-Performance Liquid Chromatography) technique, using an absorbance detector of UV/visible light at 254 nm. The extraction and subsequent analysis were based on previously published protocols [22]. The column used for the determination of GCV levels was the SunFire^®^ C18 5 μm (4.6 × 150 mm) with potassium phosphate (KH_2_PO_4_ at 0.02M, pH = 4) and methanol as eluents (99%:1%). Calibration curves were constructed.

Each patient had two blood samples (trough and peak) drawn at least 3-days post-initiation of treatment. Trough levels (Cmin) were obtained 30 min before a new dose and peak levels (Cmax) 1 h after intravenous infusion or 2 h after oral administration. No dose modifications were performed according to the results of GCV serum levels.

### 2.2. Data Collection

The following clinical data were retrieved: demographics, underlying diseases, infection type, treatment duration, indication, VGCV and GCV daily dosage and serum concentrations, laboratory findings and concomitant drugs that might potentially modify GCV serum levels. Laboratory parameters (hemoglobin, red blood cell, neutrophil and platelet counts, serum creatinine and glomerular filtration rate—GFR) were collected at baseline and during VGCV/GCV treatment. Cl_CR_ was calculated according to the Modification of Diet in Renal Disease (MDRD) equation. Patients were followed for 3-months after discharge.

Clinical outcome was classified as: (a) favorable—if there was no evidence of CMV infection [23] during prophylaxis or when a clinical and microbiological resolution was attained within 21 days of treatment and the patient did not present toxicity requiring drug discontinuation, (b) poor—in case of no clinical response, recurrence or related mortality after 21 days of treatment or if any adverse events were detected causing drug discontinuation.

Adverse events (AE) included thrombocytopenia, anemia, neutropenia, nephrotoxicity and neurotoxicity. The occurrence of thrombocytopenia and anemia during VGCV/GCV treatment was defined as a reduction of more than 30%, respectively, in platelet count or hemoglobin level from baseline. Neutropenia was defined as the presence of blood neutrophil levels lower than 1500/mL during treatment, with this low value not existing at baseline. Nephrotoxicity was defined as an increase during drug administration of 1.5 times the patient’s value of serum creatinine at baseline and neurotoxicity, as considered and assessed by the attending physician.

The Ethics Committee of the Hospital General Universitario Gregorio Marañón approved this study (study number: MICRO.HGUGM.2016-018) and all patients signed the informed consent.

### 2.3. Statistical Analysis

Median with IQR was used in the descriptive statistics for continuous variables. Categorical variables were compared by the Fisher’s exact test, and continuous variables by the Mann–Whitney test. A *p*-value < 0.05 was required to achieve statistical significance.

Univariate and multivariate logistic regression analyses were performed to identify independent predictors for poor outcome. Multivariate logistic forward conditional analysis included all variables significant at *p* ≤ 0.15 in the univariate analysis. Univariate and multivariate linear regression analyses were performed to identify independent predictors for Cmin and Cmax. Multivariate forward analyses included all variables significant at *p* ≤ 0.15 in the univariate analysis. All statistical analyses were performed using SPSS^®^ Statistics 21; IBM, Chicago, IL, USA.

## 3. Results

### 3.1. Patient Characteristics

A total of 90 patients with a diagnosis of CMV infection in the 17-month study period from June 2016 to October 2017 were screened. All the 90 patients received GCV or VGCV initially and were invited to participate. Seventy of them signed informed consent and therefore, they were included in the study. The median age was 59.2 years old and 74.3% of them were male. The median weight was 65.8 kg and the median body mass index was 24.0. Most of the patients were admitted to a medical department (70.0%). Patient characteristics are shown in Table 1/Table S1. Indication for VGCV/GCV was treatment (n = 56) and prophylaxis (n = 14). The median age-adjusted Charlson comorbidity score was 3 (1.7–5.0). The estimated GFR was normal in 41 of 70 patients (58.6%). Eight patients required extracorporeal membrane oxygenation (ECMO) (11.4%), 2 continuous renal replacement therapy (CRRT) (2.9%) and 10 were on hemodialysis (14.3%).

### 3.2. Valganciclovir and Ganciclovir Use and Their Adequacy

VGCV was used in 25 patients (35.7%) (10 as prophylaxis and 15 as treatment) and GCV in 45 patients (64.3%) (4 as prophylaxis and 41 as treatment). The main reasons for VGCV/GCV therapy were asymptomatic reactivation of CMV in 53.6% of the cases, CMV syndrome (19.6%) and CMV end-organ disease in 26.8%. Gastrointestinal disease was the most common CMV end-organ disease treated with VGCV/GCV accounting for 40% of the cases. The median duration of VGCV was 15.0 days (IQR = 11–26) and for GCV it was 14.0 days (IQR = 7–22).

The overall dose of prescribed VGCV/GCV was adequate according to guidelines [19] in only 47/70 cases (67.1%), lower than recommended in 7/70 (10%) and higher in 16/70 (22.9%). GCV was more commonly inadequately dosed than VGCV (37.8% vs 24.0%), mainly due to high doses (31.1% vs. 8.0%). As for drug indication, inadequacy was more common in treatment than in prophylaxis (35.7% vs. 21.4%), in both cases mainly due to high doses.

Abnormal GFR was the only factor related to “non-adequate” dosage found in our study (60.9% vs. 39.1%, *p* =0.038). Non-adequate VGCV/GCV dosing in patients with abnormal GFR (14 out of 29 patients = 48.3%) was mainly due to higher doses than recommended by International Guidelines, than lower doses (11/14 = 78.6% higher doses vs. 3/14 = 21.4% lower doses). Non-adequate VGCV/GCV dosing (prophylaxis and treatment altogether) was related with the presence of AE (57.1% vs. 22.4%, *p* = 0.006).

### 3.3. Valganciclovir and Ganciclovir Serum Levels and Their Variability

**Figure 1:**
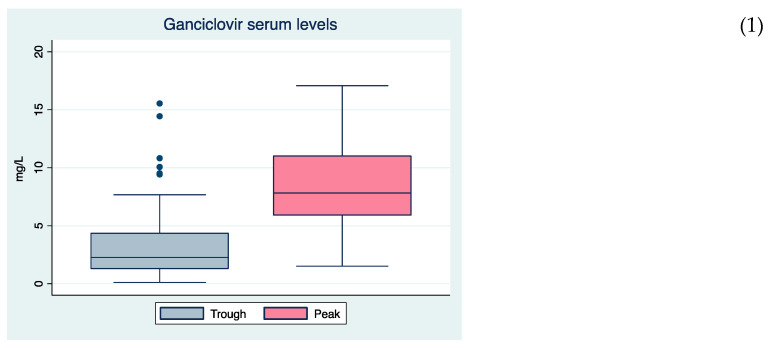

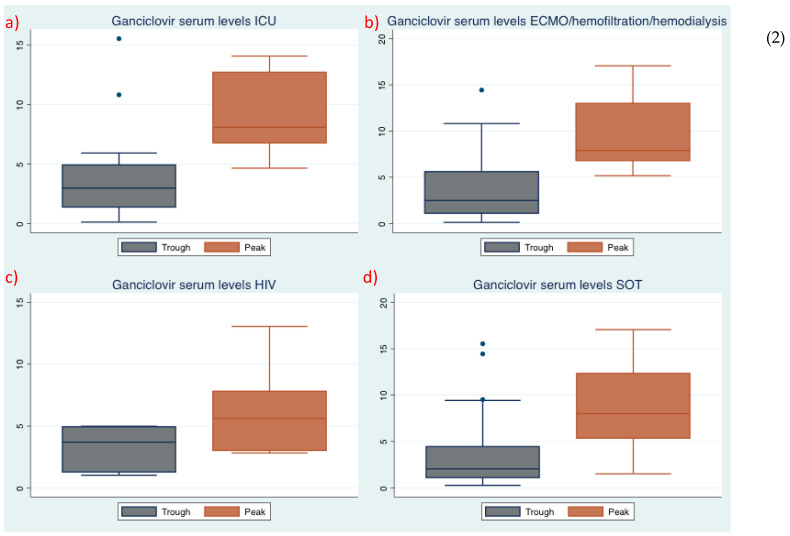
Variability in ganciclovir trough (Cmin) and peak (Cmax) serum levels (n = 70 patients) globally (1) and in subpopulations (2): (**a**) intensive care unit (ICU) patients (n = 15; prophylaxis = 2, treatment = 13), (**b**) patients under extracorporeal membrane oxygenation (ECMO)/hemofiltration/hemodialysis (n = 15; prophylaxis = 5, treatment = 10), (**c**) HIV patients (n = 8; prophylaxis = 0, treatment = 8) and (**d**) solid organ transplant recipients (n = 26; prophylaxis = 9, treatment = 17).

**Figure 2:**
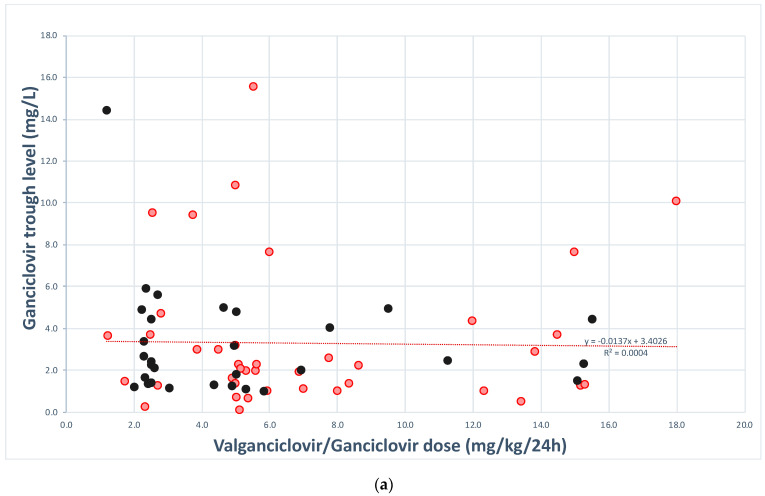

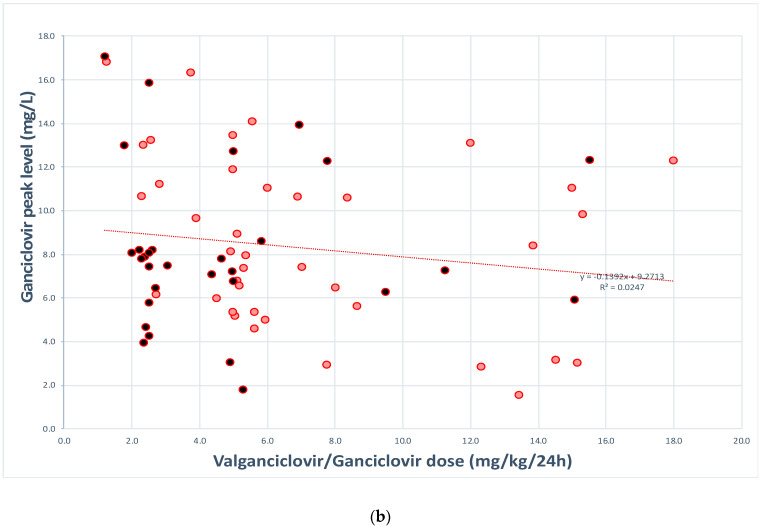
Correlation of valganciclovir/ganciclovir dose, **trough** (**a**) and **peak** (**b**) serum levels and clinical outcome. Rose dots represent patients with favorable clinical outcome and black dots represent patients with poor clinical outcome.

The median Cmin and Cmax for VGCV and GCV are shown in Table 1, Table S1 (Supplemental Material) and Figure 1. Large inter-individual variability of serum levels was observed (Figure 2), with a median trough level of 2.3 mg/L (range 0.1–15.5 mg/L) and a median peak level of 7.8 mg/L (range 1.5–17.1 mg/L). Table 2 and Figure 1 show the variability detected in small sub-populations as intensive care unit (ICU) patients, patients under ECMO/CRRT/hemodialysis, HIV patients or SOT recipients. GCV peak levels tended to be lower (*p* = 0.05) in HIV patients than in other subpopulations.

We analyzed the variables that could influence GCV serum levels (Cmin/Cmax) (Table 3A,B; Table S2A,B, Supplementary Material). Univariate and multivariate analyses identified that patients who had abnormal (low) GFR or were on hemodialysis, had higher Cmin values (Table 3A) and patients who were on hemodialysis had also higher Cmax values of VGCV/GCV (Table 3B). We were not able to confirm an association between concomitant medications (probenecid, mycophenolate mofetil, liposomal amphotericin B, trimethoprim/sulfamethoxazole, tenofovir disoproxil, tacrolimus, cyclosporine, everolimus) nor other variables and the Cmin (Table 3A) or Cmax (Table 3B).

### 3.4. Risk Factors, Valganciclovir and Ganciclovir Serum Levels and Association with Clinical Outcomes

A total of 39 patients (55.7%) achieved a favorable outcome and 31 (44.3%) a poor outcome (10 related deaths and 21 lack of clinical and microbiological resolution). No recurrence or AE needing drug discontinuation was detected.

At univariate analysis, the risk factors associated with poor outcome were age (*p* = 0.046), diabetes mellitus (*p* = 0.029), a low GFR (*p* = 0.042), hypoalbuminemia (*p* = 0.045), an inadequate initial dose of VGCV/GCV (*p* = 0.021) and a value of Cmax lower than 8.37 or greater than 11.86 mg/L (*p* = 0.042) (Table 2b, Table 1). However, after a multivariate analysis, the only variables independently associated with poor outcome were: diabetes mellitus (OR = 4.173, 95% CI = 1.147–15.179, *p* = 0.030), hypoalbuminemia (OR = 4.900, 95% CI=1.239–19.380), an inadequate initial dose of VGCV/GCV (OR = 4.673, 95% CI = 1.227–17.798, *p* = 0.038) and a value of Cmax lower than 8.37 or greater than 11.86 mg/L (OR = 9.350, 95% CI = 1.016–86.006, *p* = 0.048) (Table 1).

When we analyzed only the patients who received VGCV/GCV as treatment, the univariate analysis indicated that the risk factors associated with poor outcome were cardiac disease (*p* = 0.034), diabetes mellitus (*p* = 0.045), low GFR (*p* = 0.013), inadequate initial dose of VGCV/GCV (*p* = 0.048) and a value of Cmax lower than 8.37 or greater than 11.86 mg/L (*p* = 0.037) (Table S1, Supplemental Material). However, after a multivariate analysis, the only variables independently associated with a poor outcome were: cardiac disease (OR = 5.431, 95% CI = 1.160–25.426, *p* = 0.018), an inadequate initial dose of VGCV/GCV (OR = 3.961, 95% CI = 1.056–14.859, *p* = 0.032) and a value of Cmax lower than 8.37 or greater than 11.86mg/L (OR = 7.232, 95% CI = 0.727–71.954, *p* = 0.05) (Table S1, Supplemental Material).

We were not able to find specific Cmin values that correlated with poor outcome. However, Cmax values lower than 8.37 or greater than 11.86 mg/L did have an association with poor outcome (51.7% vs. 8.3%, *p* = 0.009) (Figure 2). Time to negative viremia was not related to trough (*p* = 0.461) or peak serum levels (*p* = 0.428).

As for AE, 2 out of 70 patients (2.9%) experienced anemia, 14 (20.0%) thrombocytopenia, and 4 (5.7%) neutropenia during VGCV/GCV treatment. Three patients developed (4.3%) nephrotoxicity and no patient presented related neurotoxicity. However, these changes could not be clearly attributable to VGCV/GCV use. There was no significant correlation between VGCV/GCV trough and peak serum levels and anemia (*p* = 0.373, *p* = 514), thrombocytopenia (*p* = 0.308, *p* = 0.618), neutropenia (*p* = 0.524, *p* = 0.125) or nephrotoxicity (*p* = 0.686, *p* = 0.817) developed during VGCV/GCV treatment.

## 4. Discussion

Approximately one-third of the patients receiving VGCV/GCV were treated with doses lower or higher than recommended by CMV guidelines. We also observed in this study that inadequate dosing of VGCV/GCV and peak levels lower than 8.37 or greater than 11.86 mg/L were related to poor outcome. Most of the patients with non-adequate doses had abnormal GFR and the presence of AEs was significantly related to non-adequate doses.

VGCV/GCV are first-line antiviral agents for prophylaxis and treatment of CMV infections given their proven clinical effectiveness [1,19]. The actual recommended doses by Kotton et al. [19] for CMV treatment are VGCV—900 mg/12 h or GCV—5 mg/kg/12 h. For CMV prophylaxis, the recommended dose of VGCV is 900 mg/24 h and for GCV—5 mg/kg/24 h. There is a consensus in most of the guidelines regarding a standard dosage but the situation is further complicated in patients with different degrees of renal failure.

Ganciclovir clearance is highly dependent on renal function, with 85% of the administered dose being renally excreted unaltered through glomerular filtration and tubular secretion [24]. For this reason, dose adjustment according to renal clearance is necessary. Hemodialysis removes 50% of blood ganciclovir [6] and also requires posology adjustment. The effect of ECMO in ganciclovir pharmacokinetics has not been well studied, although lower antiviral exposure has been reported with this technique [25]. Recommended doses vary between several sources as the International Guidelines [19], UpToDate^®^, Vademecum^®^, the drug label sheet, or Sanford^®^, regarding different creatinine clearance ranges.

Dosing intervals in our study were not always in accordance with the international guidelines, indicating a prolongation of the dosing interval from twice a day to once daily, every 2 days or 3 times a week after hemodialysis. The use of different sources for renal adjustment and non-appropriate readjustments due to unstable renal function were considered the main causes of “non-adequate” VGCV/GCV dose prescription in our hospital during the study period. The fact that MDRD was the equation used to calculate Clcr in our study but other methods are also accepted to estimate clearance in the clinical setting might account for some of the variability observed too. Dose adjustment recommendations for ganciclovir in renal failure are based on the Cockcroft–Gault equation, although in clinical practice equations such as MDRD or CKD-EPI are more commonly used to estimate renal function. Other measurements such as Cystatin C clearance when creatinine values are altered (e.g., low muscle mass, hemofiltration) have also proven to be useful in settings such as ICU [26]. Palacio-Lacambra et al. [27] found that the CKD-EPI equation correlated better with ganciclovir clearance than Cockcroft–Gault and MDRD, but further studies are necessary to establish which is the best method to be used in specific clinical situations. To address these issues, a multidisciplinary team was created in our institution to better protocolize ganciclovir and valganciclovir dosing and adjustment in different clinical scenarios.

Our results, collected systematically in a non-selected population, show a large interindividual variability of GCV serum levels occurring even in different patients receiving the same dosage. This variability in GCV serum levels has been previously noted in small populations of transplant recipients [28], HIV patients [29], and critically ill patients undergoing CRRT [4]. Fishman et al. [30] found that SOT recipients receiving GCV both as prophylaxis and treatment, with higher initial serum creatinine levels (≥3 mg/dL), had mean GCV serum levels higher than those with creatinine levels <3 mg/dL or on hemodialysis. On the same line, our multivariate analysis showed significant associations between abnormal (low) GFR and GCV trough serum levels and between patients who were on hemodialysis and peak serum levels.

GCV TDM-guided therapy has been shown to be useful in patients with renal failure [31] or before confirming GCV resistance and drug switches in HIV patients [29,32]. Campos et al. [33] found that Cmax values were associated with total dose administered but no correlation was observed between Cmin values and either dose or creatinine clearance. In our study, we were not able to find any correlation between doses administered and serum levels. However, we observed that GCV peak levels tended to be lower in HIV patients than in other subpopulations.

The therapeutic trough and peak serum levels of GCV has not been defined in CMV guidelines for any clinical entity. However, several studies suggest a therapeutic trough serum range for SOT recipients with CMV end-organ disease of 0.2–2.0 mg/L, and greater than 0.6 mg/L for HIV patients with CMV retinitis [16,29,34,35]. This target is based on in vitro observations, that establish an IC_50_ for CMV replication between 0.75–2 mg/L [36,37] but has not been validated in the clinical setting. Of note, Gimenez et al. [15] showed that persistent trough serum GCV levels greater than 0.6 mg/L were not related to CMV DNAemia clearance in stem cell transplant recipients. Perrottet et al. [11] also found variable CMV DNAemia clearance in patients with appropriate plasma levels with VGCV therapy for CMV disease in donor-positive/recipient-negative SOT recipients.

Regarding AE, only two patients in our cohort presented anemia and four, experienced neutropenia. As previously reported [28] we found no correlation between VGCV/GCV trough and peak serum levels and neutropenia. Similar to other studies [12], there was no association between VGCV/GCV trough and peak serum levels and thrombocytopenia, anemia and nephrotoxicity.

Ritchie et al. [12] did not find any association between GCV TDM and clinical efficacy or safety end-points for a proposed therapeutic range of peak and through concentrations of 3.0–12.5 mg/L and 1.0–3.0 mg/L. On the same line, in our study, we were not able to find any specific range of GCV trough serum levels related to poor outcome but peak levels lower than 8.37 mg/L or greater than 11.86 mg/L were associated with poor outcome in the multivariate analysis. Interestingly, our peak’s upper limit coincides with the one used in their institution. Patients with an increased exposure have been reported at higher risk for adverse events such as neurotoxicity and hematological toxicity [38,39,40], which could explain the worse outcomes observed in this population. To the best of our knowledge, this is the first time a minimum peak level has been associated with ganciclovir efficacy. This could suggest a concentration-dependent mechanism of action, where higher concentrations of ganciclovir are necessary to successfully inhibit CMV replication in vivo.

Our work shows the clinical use of VGCV and GCV in patients with different conditions and real-life results from an HPLC novel technique in our institution. This study adds our experience of VGCV/GCV use to previously published literature along with the TDM value, also evaluating specific clinical scenarios such as HIV patients, patients at ICU, under ECMO, hemofiltration or hemodialysis and solid organ transplant recipients. However, it has several limitations. The fact that a high number of patients were receiving VGCV/GCV as prophylaxis, the small sample size and lack of power for some comparisons are probably the most relevant.

## 5. Conclusions

High variability in VGCV/GCV dosing and adjustment was found in our study, which led us to implement new protocols to improve standardization. Large inter-individual variability of serum levels was observed, and peak levels lower than 8.37 or greater than 11.86 mg/L and inadequate dosing, were related to poor outcome. Our data also set a basis to design a pharmacokinetic model in a higher group of patients with different serum drug determinations per patient needed to obtain more accurately the AUC and other PK/PD parameters for GCV exposure [41]. Further studies must be performed to confirm these results and to conclusively establish if VGCV/GCV TDM could be useful to improve outcomes and minimize drug-related toxicity and the emergence of CMV resistance in specific populations, like renal transplant and HSCT recipients [17,42].

## Figures and Tables

**Table 1:** Poor outcome risk factors: univariate and multivariate analysis.

GLOBAL		Univariate Analysis			Multivariate Analysis		
Characteristics	Global (n = 70)	Poor Clinical Outcome (n = 31)	Favorable Clinical Outcome (n = 39)	*p* Value	OR	95 % CI	*p* Value
**General characteristics**							
Age (years), median (IQR)	59.2 (46.5–69.7)	64.9 (52.8–72.6)	53.5 (42.4–65.8)	0.046			
Sex, male, n (%)	52 (74.3)	22 (71)	30 (76.9)	0.594			
Weight (kg), median (IQR)	65.8 (56.0–75.12)	68.6 (57.6–78.6)	65 (55–75)	0.493			
Body mass index (mg/kg^2^), median (IQR)	24.0 (20.6–27.7)	25.7 (22.8–28.7)	23 (19.4–26.8)	0.105			
**Race, n (%)**				1.0			
Caucasian	60 (85.7)	27 (87.1)	33 (84.6)				
Other	10 (14.3)	4 (12.9)	6 (15.4)				
**Department of admission, n (%)**				0.163			
Medical	47 (67.1)	18 (58.1)	29 (74.4)				
Surgical	8 (11.4)	3 (9.7)	5 (12.8)				
ICU	15 (21.4)	10 (32.3)	5 (12.8)				
**Underlying disease, n (%)**							
Cardiac disease	21 (30.0)	12 (38.7)	9 (23.1)	0.194			
Diabetes mellitus	20 (28.6)	13 (41.9)	7 (17.9)	0.029	4.173	1.147–15.179	0.030
Solid tumor	8 (11.4)	4 (12.9)	4 (10.3)	1.0			
Chronic renal failure	20 (28.6)	11 (35.5)	9 (23.1)	0.295			
Liver disease	13 (18.6)	7 (22.6)	6 (15.4)	0.541			
HIV infection	8 (11.4)	3 (9.7)	5 (12.8)	0.726			
Neurologic disease	7 (10.0)	2 (6.5)	5 (12.8)	0.452			
Chronic Obstructive Pulmonary Disease	5 (7.1)	3 (9.7)	2 (5.1)	0.649			
Solid Organ Transplantation	26 (37.1)	12 (38.7)	14 (35.9)	1.0			
Cardiac transplant	10 (14.3)	5 (16.1)	5 (12.8)	0.741			
Liver transplant	11 (15.7)	6 (19.4)	5 (12.8)	0.520			
Renal transplant *	6 (8.6)	2 (6.5)	4 (10.3)	0.687			
Haematologic neoplasia	7 (10.0)	2 (6.5)	5 (12.8)	0.452			
Psychiatric disease	5 (7.1)	2 (6.5)	3 (7.7)	1.0			
Other	3 (4.3)	2 (6.5)	1 (2.6)	0.580			
**Charlson’s index, median (IQR)**	3 (1.7–5.0)	4 (3-6)	3 (1–5)	0.098			
**McCabe index, n (%)**				0.408			
Non-fatal	40 (57.1)	16 (51.6)	24 (61.5)				
Ultimately fatal	21 (30.0)	9 (29.0)	12 (30.8)				
Rapidly fatal	9 (12.9)	6 (19.4)	3 (7.7)				
**eGFR (mL/min/1.73 m^2^), n (%)**				0.042			
Normal (≥60)	41 (58.6)	14 (45.2)	27 (69.2)				
Low (<60)	29 (41.4)	17 (54.8)	12 (30.8)				
**Hypoalbuminemia (<3.4 g/dL)**	46 (65.7)	24 (80.0)	22 (56.4)	0.045	4.900	1.239–19.380	0.024
**Hemodialysis, n (%)**	10 (14.3)	5 (16.1)	5 (12.8)	0.741			
**ECMO, n (%)**	8 (11.4)	4 (12.9)	4 (10.3)	1.0			
**Hemofiltration, n (%)**	2 (2.9)	2 (6.5)	0	0.193			
**Type of treatment VGCV/GCV, n (%)**							
Prophylaxis	14/70 (20)	0	14 (35.9)	0.001			
Targeted	56/70 (80)	31 (100)	25 (64.1)				
Asymptomatic reactivation	30/56 (53.6)	18 (58.1)	12 (30.8)	0.029			
CMV syndrome	11/56 (19.6)	6 (19.4)	5 (12.8)	0.520			
CMV end-organ disease	15/56 (26.8)	7 (22.6)	8 (20.5)	1.0			
**Main indications for valganciclovir/ganciclovir CMV end-organ disease, n (%)**							
Pneumonia	5/15 (33.3)	3 (9.7)	2 (5.1)	0.649			
Retinitis	1/15 (6.7)	0	1 (2.6)	1.0			
Hepatitis	0	0	0	NA			
CNS disease	2/15 (13.3)	0	2 (5.1)	0.499			
Nephritis	0	0	0	NA			
Gastrointestinal disease	6/15 (40)	3 (9.7)	3 (7.7)	1.0			
Myocarditis	0	0	0	NA			
Cystitis	0	0	0	NA			
Cholangitis	1/15 (6.7)	1 (3.2)	0	0.443			
Pancreatitis	0	0	0	NA			
Basal CMV viral load, median (IQR) **	1361 (496.2–9322.5)	3995 (558–10034)	742 (404.5–6797)	0.132			
**Time (days) to negative viremia Median (IQR)**	12.5 (4–21)	17 (9–23.5)	7.5 (3–13)	0.005			
**ICU, days, median (IQR)**	31.0 (15.0–63.0)	29 (20–50)	63 (12–133.5)	0.574			
**Hospital stay, days, median (IQR)**	48.5 (23.0–92.5)	51 (27–106)	44 (15–89)				
**Valganciclovir/ganciclovir use**							
Duration of prophylaxis/treatment (days), median (IQR)	14.5 (8–22)	17 (9–22)	14 (8–21)	0.663			
Dose adequacy, n (%)							
Adequate dose	47/70 (67.1)	16 (51.6)	31 (79.5)	0.021	4.673	1.227–17.798	0.024
Non-adequate (infradoses)	7/70 (10.0)	5 (16.1)	2 (5.1)	0.228			
Non-adequate (supradoses)	16/70 (22.9)	10 (32.3)	6 (15.4)	0.151			
**Ganciclovir serum level**							
Cmin (mg/L), median (IQR)	2.3 (1.3–4.4)	2.3 (1.4–4.5)	2.2 (1.2–3.7)	0.493			
Cmax (mg/L), median (IQR)	7.8 (5.8–11.1)	7.6 (6.2–9.5)	8.1 (5.4–11.2)	0.780			
Cmax <8.37 mg/L or >11.86 mg/L, n (%)	12 (17.1)	1 (3.2)	11 (28.2)	0.009	9.350	1.016–86.006	0.048
**Concomitant medications, n (%)**							
Probenecid	1 (1.4)	0	1 (2.6)	1.0			
Mycophenolate mofetil	26 (37.1)	12 (38.7)	14 (35.9)	1.0			
Zidovudine	0	0	0	NA			
Stavudine	0	0	0	NA			
Didanosine	0	0	0	NA			
Imipenem	0	0	0	NA			
Amphotericin B	1 (1.4)	1 (3.2)	0	0.443			
Trimethoprim/sulfamethoxazole	33 (47.1)	14 (45.2)	19 (48.7)	0.813			
Hydroxyurea	0	0	0	NA			
Pentamidine	0	0	0	NA			
Flucytosine	0	0	0	NA			
Vincristine	1 (1.4)	1 (3.2)	0	0.443			
Vinblastine	1 (1.4)	1 (3.2)	0	0.443			
Doxorubicin	0	0	0	NA			
Dapsone	0	0	0	NA			
Tenofovir disoproxil	4 (5.7)	2 (6.5)	2 (5.1)	1.0			
Foscarnet	0	0	0	NA			
Cidofovir	0	0	0	NA			
Tacrolimus	27 (38.6)	11 (35.5)	16 (41)	0.805			
Cyclosporine	5 (7.1)	1 (3.2)	4 (10.3)	0.374			
Everolimus	3 (4.3)	0	3 (7.7)	0.249			

ECMO: extracorporeal membrane oxygenation. eGFR (MDRD): estimated glomerular filtration rate (Modification of Diet in Renal Disease equation). ICU: intensive care unit. * One patient had renal and liver transplant, ** Only in non-prophylaxis cases, NA. Not available.

**Table 2:** Serum valganciclovir/ganciclovir trough and peak levels in the different subpopulations.

Patients, n (Prophylaxis/Treatment)	Ganciclovir Serum Levels					
	Prophylaxis		Treatment		*p* Trough	*p* Peak
	Trough, median (mg/L, IQR)	Peak, median (mg/L, IQR)	Trough, median (mg/L, IQR)	Peak, median (mg/L, IQR)		
Global cohort, n = 70 (14/56)	1.7 (1.1–3.0)	7.9 (5.3–11.2)	2.4 (1.4–4.5)	7.8 (5.9–11.2)		
Intensive care unit, n = 15 (2/13)	NA *	NA *	2.7 (1.3–4.9)	7.9 (6.8–11.1)	0.336	0.267
ECMO/hemofiltration/hemodialysis, n = 15 (5/10)	1.1 (0.5–2.3)	10.6 (6.3–12.4)	3.2 (1.7–7.2)	7.9 (6.8–14.3)	0.924	0.101
HIV, n = 8 (0/8)	NA	NA	3.0 (1.5–4.8)	5.6 (3.0–7.8)	0.522	0.053
Solid organ transplant, n = 26 (9/17)	1.2 (0.9–5.3)	7.4 (5.7–12.0)	2.7 (1.3–5.0)	8.2 (4.7–12.8)	0.784	0.603

* Only 2 values. ECMO: extracorporeal membrane oxygenation. NA. Not available.

**Table 3:** Univariate and multivariate analysis of variables associated with Cmin (A) and Cmax (B) of valganciclovir/ ganciclovir (n = 70).

**A**. Univariate and multivariate analysis of variables associated with Cmin of valganciclovir/ganciclovir (n = 70)				
**GLOBAL**	**Univariate Analysis**		**Multivariate Analysis**	
**Variable**	**Unstandardized β-Coefficient** **(95% CI)**	***p***	**Unstandardized β-Coefficient** **(95% CI)**	***p***
Age (years)	0.022 (−0.015 to 0.059)	0.239		
Sex	−0.420 (−2.128 to 1.288)	0.625		
Weight (kg)	0.004 (−0.032 to 0.039)	0.842		
Body mass index (m^2^/kg)	−0.006 (−0.122 to 1.110)	0.922		
eGFR (MDRD) abnormal (low)	1.687 (0.225 to 3.150)	0.024	1.730 (0.3 to 3.160)	0.018
Dose (mg/kg)	−0.012 (−0.092–0.068)	0.765		
Hemodialysis	1.605 (−0.496 to 3.707)	0.132		
ECMO	−0.068 (−2.418 to 2.283)	0.954		
Hemofiltration	−1.283 (−5.761 to 3.195)	0.569		
Beginning in ICU	1.023 (−0.782 to 2.829)	0.262		
Solid organ transplant	2.978 (2.044–3.911)	0.239		
HIV	−0.243 (−2.592–2.107)	0.837		
Hypoalbuminemia	0.287 (−1.316 to 1.889)	0.722		
**Co-treatment with:**				
Probenecid	−0.351 (−6.652 to 5.950)	0.912		
Mycophenolate mofetil	1.185 (−0.336 to 2.705)	0.125		
Amphotericin B	−2.137 (−0.417 to 4.144)	0.500		
Trimethoprim/sulfamethoxazole	0.773 (−0.714 to 2.259)	0.303		
Tenofovir disoproxil	−0.327 (−3.548 to 2.893)	0.840		
Tacrolimus	0.949 (−0.570 to 2.468)	0.217		
Cyclosporine	−0.847 (−3.743 to 2.050)	0.562		
Everolimus	−1.553 (−5.226 to 2.120)	0.402		
**Charlson**	−0.024 (−0.348 to 0.301)	0.885		
**McCabe**	−0.475 (−1.522 to 0.572)	0.368		
**B.** Univariate and multivariate analysis of variables associated with **Cmax** of valganciclovir/ganciclovir (n = 70)				
**GLOBAL**	**Univariate Analysis**		**Multivariate Analysis**	
**Variable**	**Unstandardized β-Coefficient** **(95% CI)**	***p***	**Unstandardized β-Coefficient** **(95% CI)**	***p***
Age (years)	0.002 (−0.043 to 0.048)	0.923		
Sex	1.024 (−1.075 to 3.124)	0.334		
Weight (kg)	−0.020 (−0.063 to 0.023)	0.364		
Body mass index (m^2^/kg)	−0.031 (−0.172 to 0.109)	0.659		
eGFR (MDRD) abnormal (low)	1.898 (0.111 to 3.685)	0.038		
Dose (mg/kg)	−0.037 (−0.137–0.063)	0.464		
Hemodialysis	3.173 (0.703 to 5.643)	0.013	3.173 (0.703 to 5.643)	0.013
ECMO	1.512 (−1.310 to 4.334)	0.289		
Hemofiltration	2.037 (−3.372 to 7.445)	0.455		
Beginning in ICU	0.995 (−1.201 to 3.190)	0.369		
Solid organ transplant	0.648 (−1.225-2.522)	0.492		
HIV	−2.719 (−5.664-0.225)	0.070		
Hypoalbuminemia	0.253 (−1.655 to 2.162)	0.792		
**Co-treatment with:**				
Probenecid	1.256 (−6.362 to 8.874)	0.743		
Mycophenolate mofetil	0.324 (−1.554 to 2.203)	0.732		
Amphotericin B	−0.327 (−7.951 to 7.297)	0.932		
Trimethoprim/sulfamethoxazole	−0.765 (−2.580 to 1.050)	0.403		
Tenofovir disoproxil	−3.989 (−8.350 to 0.372)	0.072		
Tacrolimus	0.923 (−0.931 to 2.776)	0.324		
Cyclosporine	−0.917 (−4.424 to 2.591)	0.604		
Everolimus	1.277 (−3.181 to 5.734)	0.569		
**Charlson**	−0.115 (−0.514 to 0.284)	0.567		
**McCabe**	−0.978 (−2.235 to 0.280)	0.126		

ECMO: extracorporeal membrane oxygenation. eGFR (MDRD): estimated glomerular filtration rate (Modification of Diet in Renal Disease equation). ICU: intensive care unit.

## Data Availability

Data is contained within the article or supplementary material.

## Supplementary Materials

The following are available online at https://www.mdpi.com/2079-6382/10/1/77/s1, Table S1. Poor outcome risk factors: univariate and multivariate analysis. Only patients who received valganciclovir/ganciclovir as treatment (n=56), Table S2A. Univariate and multivariate analysis of variables associated with Cmin of valganciclovir/ ganciclovir administered as prophylaxis or treatment, Table S2B. Univariate and multivariate analysis of variables associated with Cmax of valganciclovir/ ganciclovir administered as prophylaxis or treatment.
