# Supplementary material for: Valganciclovir—Ganciclovir Use and Systematic Therapeutic Drug Monitoring. An Invitation to Antiviral Stewardship
Source: Antibiotics (Basel). 2021 Jan 15;10(1):77. doi: 10.3390/antibiotics10010077 (PMC7831032; doi:10.3390/antibiotics10010077)
Supplement: Supplementary file 1 [file antibiotics-10-00077-s001.pdf]

Supplementary material

Table S1. Poor outcome risk factors: univariate and multivariate analysis. Only patients who received valganciclovir/ganciclovir as **treatment** (n=56)

TREATMENT			**Univariate analysis**			**Multivariate analysis**	
**Characteristics**	Global (n=56)	Poor clinical outcome (n=31)	Favorable clinical outcome (n=25)	*p* value	OR	95 % CI	*p* value
**General characteristics**							
Age (years), median (IQR)	60.9 (48.0-	64.9 (52.8-72.6)	53.5 (43.8-71.1)	0.182			
Sex, male, n (%)	41 (73.2)	22 (71.0)	19 (76.0)	0.767			
Weight (kg), median (IQR)	65.8 (57.7-	68.6 (57.6-78.6)	65.0 (57.0-72.5)	0.383			
Body mass index (mg/kg^2), median (IQR)	23.9 (20.9-28.1)	25.7 (22.8-28.7)	22.9 (20.0-26.6)	0.130			
**Race, n (%)**				1.0			
Caucasian	48 (85.7)	27 (87.1)	21 (84.0)				
Other	8 (14.3)	4 (12.9)	4 (16.0)				
**Department of admission, n (%)**				0.191			
Medical	36 (64.3)	18 (58.1)	18 (72.0)				
Surgical	7 (12.5)	3 (9.7)	4 (16.0)				
ICU	13 (23.2)	10 (32.3)	3 (12.0)				
**Underlying disease, n (%)**							
Cardiac disease	15 (26.8)	12 (38.7)	3 (12.0)	**0.034**	5.431	(1.160-25.426)	**0.018**
Diabetes mellitus	17 (30.4)	13 (41.9)	4 (16.0)	**0.045**			
Solid tumor	7 (12.5)	4 (12.9)	3 (12.0)	1.0			
Chronic renal failure	14 (25.0)	11 (35.5)	3 (12.0)	0.063			
Liver disease	11 (19.6)	7 (22.6)	4 (16.0)	0.737			
HIV infection	8 (14.3)	3 (9.7)	5 (20.0)	0.445			
Neurologic disease	6 (10.7)	2 (6.5)	4 (16.0)	0.391			
Chronic Obstructive Pulmonary Disease	5 (8.9)	3 (9.7)	2 (8.0)	1.0			
Solid Organ Transplantation							
Cardiac transplant	5 (8.9)	5 (16.1)	0 (0)	0.058			
Liver transplant	10 (17.9)	6 (19.4)	4 (16.0)	1.0			
Renal transplant*	3 (5.4)	2 (6.5)	1 (4.0)	1.0			
Haematologic neoplasia	5 (8.9)	2 (6.5)	3 (12.0)	0.647			
Psychiatric disease	4 (7.1)	2 (6.5)	2 (8.0)	1.0			
Other	3 (5.4)	2 (6.5)	1 (4.0)	1.0			
**Charlson´s index, median (IQR)**	3.5 (2.0-5.0)	4 (3-6)	3 (1.5-5)	0.217			
**McCabe index, n (%)**				0.718			
Non-fatal	29 (51.8)	16 (51.6)	13 (52.0)				
Ultimately fatal	18 (32.1)	9 (29.0)	9 (36.0)				
Rapidly fatal	9 (16.1)	6 (19.4)	3 (12.0)				
**eGFR (mL/min/1.73m^2), n (%)**				**0.013**			

Normal (≥60)	34 (60.7)	14 (45.2)	20 (80.0)				
Low (<60)	22 (39.3)	17 (54.8)	5 (20.0)				
**Hypoalbuminemia (<3.4 g/dL)**	38 (69.1)	24 (80.0)	14 (56.0)	0.080			
**Hemodialysis, n (%)**	7 (12.5)	5 (16.1)	2 (8.0)	0.443			
**ECMO, n (%)**	5 (8.9)	4 (12.9)	1 (4.0)	0.367			
**Hemofiltration, n (%)**	2 (3.6)	2 (6.5)	0 (0)	0.497			
**Type of treatment VGCV/GCV, n (%)**							
Asymptomatic reactivation	30 (53.6)	18 (58.1)	12 (48.0)	0.591			
CMV syndrome	11 (19.6)	6 (19.4)	5 (20.0)	1.0			
CMV end-organ disease	15 (26.8)	7 (22.6)	8 (32.0)	0.547			
**Main indications for valganciclovir/ganciclovir CMV end-organ disease, n (%)**[1]							
Pneumonia	5 (8.9)	3 (9.7)	2 (8.0)	1.0			
Retinitis	1 (1.8)	0	1 (4.0)	0.446			
Hepatitis	0	0	0	NA			
CNS disease	2 (3.6)	0	2 (8.0)	0.195			
Nephritis	0	0	0	NA			
Gastrointestinal disease	6 (10.7)	3 (9.7)	3 (12.0)	1.0			
Myocarditis	0	0	0	NA			
Cystitis	0	0	0	NA			
Cholangitis	1 (1.8)	1 (3.2)	0	1.0			
Pancreatitis	0	0	0	NA			
**Basal CMV viral load, median (IQR)**	1361 (496.2-9322.5)	3995 (558-10034)	742 (404.5-6797)	0.133			
**Time (days) to negative viremia Median (IQR)**	13.0 (4.7-21.0)	17 (9-23.5)	9.5 (3.7-13.5)	0.017			
**ICU, days, median (IQR)**	41 (23.5-	29.0 (22.0-50.0)	73 (68-133.5)	0.024			
**Hospital stay, days, median (IQR)**	52.5 (24.0-	51 (27-106)	58 (22-90)	0.873			
**Valganciclovir/ ganciclovir use**							
Duration of prophylaxis/treatment (days), median (IQR)	14 (7.25-21.0)	17 (9-22)	14 (6.5-19.5)	0.229			
Dose adequacy, n (%)							
Adequate dose	36 (64.3)	16 (51.6)	20 (80.0)	**0.048**	3.961	(1.056-14.859)	**0.032**
Non adequate (infradoses)	7 (12.1)	5 (16.1)	2 (8.0)	0.443			
Non adequate (supradoses)	13 (23.2)	10 (32.3)	3 (12.0)	0.112			
**Ganciclovir serum level**							
Cmin (mg/L), median (IQR)	2.4 (1.4-4.5)	2.3 (1.4-4.5)	2.6 (1.3-4.5)	0.977			
Cmax (mg/L), median (IQR)	7.8 (5.9-	7.6 (6.2-9.5)	8.1 (5.5-11.8)	0.837			
Cmax<8.37 mg/L or >11.86 mg/L, n (%)	7 (12.5)	1 (3.2)	6 (24.0)	**0.037**	7.232	(0.727-71.954)	**0.05**
**Concomitant medications, n (%)**							
Probenecid	1 (1.8)	0	1 (4.0)	0.446			

Mycophenolate mofetil	17 (30.4)	12 (38.7)	5 (20.0)	0.155
Zidovudine	0	0	0	NA
Stavudine	0	0	0	NA
Didanosine	0	0	0	NA
Imipenem	0	0	0	NA
Amphotericin B	1 (1.8)	1 (3.2)	0	1.0
Trimethoprim/sulfamethoxazole	23 (41.1)	14 (45.2)	9 (36.0)	0.589
Hydroxyurea	0	0	0	NA
Pentamidine	0	0	0	NA
Flucytosine	0	0	0	NA
Vincristine	1 (1.8)	1 (3.2)	0	1.0
Vinblastine	1 (1.8)	1 (3.2)	0	1.0
Doxorubicin	0	0	0	NA
Dapsone	0	0	0	NA
Tenofovir disoproxil	4 (7.1)	2 (6.5)	2 (8.0)	1.0
Foscarnet	0	0	0	NA
Cidofovir	0	0	0	NA
Tacrolimus	17 (30.4)	11 (35.5)	6 (24.0)	0.395
Cyclosporine	4 (7.1)	1 (3.2)	3 (12.0)	0.314
Everolimus	2 (3.6)	0	2 (8.0)	0.195

ECMO: extracorporeal membrane oxygenation. eGFR (MDRD): estimated glomerular filtration rate (Modification of Diet in Renal Disease equation). ICU: intensive care unit

Table S2A. Univariate and multivariate analysis of variables associated with **Cmin** of valganciclovir/ ganciclovir administered as prophylaxis or treatment

**PROPHYLAXIS**	**Univariate analysis**		**Multivariate analysis**	
**Variable**	**Unstandardized β-coefficient (95% CI)**	**p**	**Unstandardized β-coefficient (95% CI)**	**p**
Age (years)	0.038 (-0.066-0.143)	0.440		
Sex	-1.510 (-7.378-4.359)	0.585		
Weight (kg)	0.040 (-0.052-0.132)	0.361		
Body mass index	0.056 (-0.432-0.543)	0.807		
eGFR (MDRD)	2.631 (-1.958-7.221)	0.235		
Dose (mg/kg)	-0.052 (-0.334-0.230)	0.696		
Hemodialysis	-2.872 (-8.536-2.793)	0.291		
ECMO	-1.942 (-7.761-3..876)	0.481		
Hemofiltration	NA			
Beginning in ICU	NA			
Solid organ transplant	1.594 (-3.397-6.586)	0.500		
HIV	NA			
Hypoalbuminemia	0.247 (-4.680-5.174)	0.915		
**Co-treatment with:**				
Probenecid	NA			
Mycophenolate mofetil	2.496 (-2.346-7.339)	0.283		
Amphotericin B	NA			
Trimethoprim/sulfamet	2.776 (-2.334-7.886)	0.259		
Tenofovir disoproxil	NA			
Tacrolimus	2.472 (-2.700-7.643)	0.318		
Cyclosporine	-1.877 (-11.275-7.521)	0.671		
Everolimus	-1.812 (-11.215-7.591)	0.682		
**Charlson**	0.262 (-0.740-1.263)	0.580		
**McCabe**	-1.026 (-6.936-4.884)	0.712		

**TREATMENT**	**Univariate analysis**		**Multivariate analysis**	
**Variable**	**Unstandardized β-coefficient (95% CI)**	**p**	**Unstandardized β-coefficient (95% CI)**	**p**
Age (years)	0.015 (-0.027-0.057)	0.468		
Sex	-0.207 (-1.965-1.551)	0.814		
Weight (kg)	-0.013 (-0.053-0.027)	0.516		
Body mass index	-0.017 (-0.133-0.099)	0.767		
eGFR (MDRD)	1.488 (-0.054-3.030)	0.058		
Dose (mg/kg)	-0.005 (-0.088-0.077)	0.9		
Hemodialysis	3.409 (1.245-5.573)	**0.003**	3.409 (1.245-5.573)	**0.003**
ECMO	1.009 (-1.709-3.727)	0.460		
Hemofiltration	-1.366 (-5.547-2.815)	0.515		
Beginning in ICU	NA			
Solid organ transplant	0.939 (-0.735-2.614)	0.266		
HIV	-0.333 (-2.557-1.891)	0.765		
Hypoalbuminemia	-0.264 (-1.979-1.450)	0.758		
**Co-treatment with:**				
Probenecid	-0.425 (-6.305-5.456)	0.885		

Mycophenolate mofetil	1.071 (-0.598-2.740)	0.204
Amphotericin B	-2.217 (-8.067-3.634)	0.451
Trimethoprim/sulfamet	0.498 (-1.079-2.075)	0.529
Tenofovir disoproxil	-0.409 (-3.431-2.614)	0.787
Tacrolimus	0.871 (-0.806-2.548)	0.302
Cyclosporine	-0.589 (-3.609-2.431)	0.697
Everolimus	-1.366 (-5.547-2.815)	0.515
**Charlson**	-0.116 (-0.460-0.228)	0.503
**McCabe**	-0.520 (-1.560-0.520)	0.321

ECMO: extracorporeal membrane oxygenation. eGFR (MDRD): estimated glomerular filtration rate (Modification of Diet in Renal Disease equation). ICU: intensive care unit

Table S2B. Univariate and multivariate analysis of variables associated with **Cmax** of valganciclovir/ ganciclovir administered as prophylaxis or treatment

**PROPHYLAXIS**	**Univariate analysis**		**Multivariate analysis**	
**Variable**	**Unstandardized β-coefficient (95% CI)**	**p**	**Unstandardized β-coefficient (95% CI)**	**p**
Age (years)	-0.079 [-0.148-(-0.010)]	**0.028**	-0.063 [(-0.125--(-0.002)]	**0.045**
Sex	1.316 (-3.322-5.955)	0.548		
Weight (kg)	-0.066 [-0.129-(-0.003)]	**0.043**		
Body mass index	-0.335 [-0.660-(-0.010)]	**0.045**		
eGFR (MDRD)	0.146 (-3.720-4.011)	0.936		
Dose (mg/kg)	0.023 (-0.201-0.248)	0.824		
Hemodialysis	1.316 (-3.322-5.955)	0.548		
ECMO	2.814 (-1.553-7.181)	0.186		
Hemofiltration	NA			
Beginning in ICU	5.162 (0.691-9.632)	**0.027**	4.155 (0.132-8.177)	**0.044**
Solid organ transplant	0.189 (-3.844-4.222)	0.920		
HIV	NA			
Hypoalbuminemia	-1.442 (-5.242-2.357)	0.424		
**Co-treatment with:**				
Probenecid	NA			
Mycophenolate mofetil	-0.797 (-4.800-3.207)	0.672		
Amphotericin B	NA			
Trimethoprim/sulfamet	0.876 (-3.368-5.120)	0.661		
Tenofovir disoproxil	NA			
Tacrolimus	0.228 (-4.049-4.506)	0.909		
Cyclosporine	1.351 (-6.108-8.809)	0.700		
Everolimus	2.180 (-5.201-9.561)	0.532		
**Charlson**	-0.242 (-1.031-0.548)	0.517		
**McCabe**	-1.297 (-5.938-3.344)	0.554		

**TREATMENT**	**Univariate analysis**		**Multivariate analysis**	
**Variable**	**Unstandardized β-coefficient (95% CI)**	**p**	**Unstandardized β-coefficient (95% CI)**	**p**
Age (years)	0.035 (-0.022-0.091)	0.229		
Sex	0.967 (-1.476-3.411)	0.431		
Weight (kg)	0.0 (-0.054-0.055)	0.988		
Body mass index	0.012 (-0.147-0.170)	0.881		
eGFR (MDRD)	2.364 (0.278-4.451)	**0.027**		
Dose (mg/kg)	-0.050 (-0.166-0.066)	0.391		
Hemodialysis	3.906 (0.879-6.933)	**0.012**	3.906 (0.879-6.933)	**0.012**
ECMO	0.842 (-2.875-4.560)	0.651		
Hemofiltration	2.088 (-3.604-7.779)	0.465		
Beginning in ICU	0.303 (-2.217-2.822)	0.811		
Solid organ transplant	0.789 (-1.518-3.096)	0.496		
HIV	-2.761 (-5.883-0.361)	0.082		
Hypoalbuminemia	0.868 (-1.470-3.205)	0.460		
**Co-treatment with:**				
Probenecid	1.295 (-6.711-9.302)	0.747		

Mycophenolate mofetil	0.612 (-1.699-2.923)	0.597
Amphotericin B	-0.294 (-8.308-7.721)	0.942
Trimethoprim/sulfamet	-1.213 (-3.358-0.932)	0.262
Tenofovir disoproxil	-4.0 (-8.584-0.585)	0.086
Tacrolimus	1.171 (-1.124-3.465)	0.311
Cyclosporine	-1.484 (-5.587-2.620)	0.472
Everolimus	0.817 (-4.899-6.532)	0.776
**Charlson**	-0.075 (-0.557-0.408)	0.758
**McCabe**	-0.995 (-2.408-0.418)	0.164

ECMO: extracorporeal membrane oxygenation. eGFR (MDRD): estimated glomerular filtration rate (Modification of Diet in Renal Disease equation). ICU: intensive care unit
